# Comparison of Ligation of Intersphincteric Fistula Tract and Conventional Fistulectomy in the Management of Perianal Fistula

**DOI:** 10.7759/cureus.113807

**Published:** 2026-08-02

**Authors:** Dhivya Kumar, Vikas C Kawarat, Prabhakar Padmanabhan, Abishek Kumar Ashok Kumar, Sahasyaa Adalarasan

**Affiliations:** 1 General Surgery, Madras Medical College, Chennai, IND; 2 Medicine, Madras Medical College, Chennai, IND

**Keywords:** anal fistula, fistulectomy, lift, perianal fistula, randomized controlled trial, sphincter preservation

## Abstract

Background

Perianal fistula is a chronic anorectal disorder characterized by an epithelialized tract extending between the anal canal and the perianal skin, most frequently developing after cryptoglandular abscess formation. Patients commonly experience persistent discharge, pain, recurrent infections, and impaired quality of life. Although conventional fistulectomy remains an established treatment option, it may result in greater postoperative discomfort, slower wound healing, and potential impairment of continence. The ligation of intersphincteric fistula tract (LIFT) technique has gained acceptance as a sphincter-preserving procedure intended to minimize these complications while maintaining satisfactory treatment outcomes.

Aim

This study aimed to compare the clinical outcomes of the sphincter-preserving LIFT procedure with conventional fistulectomy in patients with perianal fistula by evaluating postoperative pain, wound infection, wound healing, postoperative fecal incontinence, and recurrence.

Methods

This prospective, randomized controlled trial was conducted in the Department of General Surgery at a tertiary care institution. Thirty patients with perianal fistula were randomly allocated in a 1:1 ratio to undergo either the LIFT procedure (n = 15) or conventional fistulectomy (n = 15). Outcomes assessed during follow-up included postoperative pain, wound infection, wound healing, postoperative fecal incontinence, and recurrence. Continuous variables were compared using the Mann-Whitney U test, while categorical variables were analysed using Fisher's exact test. Statistical significance was defined as a two-sided p value <0.05.

Results

Compared with conventional fistulectomy, the LIFT procedure was associated with significantly lower postoperative pain scores (median Visual Analogue Scale score: 2 vs. 5; Mann-Whitney U test, p < 0.001), lower rates of postoperative wound infection (6.7% vs. 46.7%; Fisher's exact test, p = 0.035), and improved postoperative wound healing (93.3% vs. 53.3%; Fisher's exact test, p = 0.035). Although postoperative recurrence (6.7% vs. 26.7%) and fecal incontinence (0.0% vs. 6.7%) occurred less frequently following the LIFT procedure, these differences were not statistically significant.

Conclusion

The LIFT procedure was associated with lower postoperative pain, fewer wound infections, and improved wound healing compared with conventional fistulectomy. Although lower rates of postoperative recurrence and fecal incontinence were observed following LIFT, these differences were not statistically significant. Larger multicenter studies with longer follow-up are required to confirm these findings.

## Introduction

Perianal fistula is an abnormal tract lined by granulation tissue that connects an opening on the perianal skin (external opening) to another opening inside the anal canal or the lower rectum (internal opening) [1,2]. It commonly develops as a chronic sequela of anorectal abscess secondary to cryptoglandular infection [3,4]. Symptoms include chronic purulent discharge, pain, local irritation and recurrent abscess formation with intermittent spontaneous decompression. Internal openings are usually solitary while external openings can be single or multiple. Fistula-in-ano is associated with significant morbidity, discomfort, social embarrassment, and impairment of quality of life due to persistent discharge and repeated infections [5]. Descriptions of anal fistulas date back over two millennia, with Hippocrates recommending the use of a seton as early as 430 BC. During the late nineteenth and early twentieth centuries, Goodsall and Milligan-Morgan conducted extensive research and developed various treatment methodologies [6,7]. The classification system introduced by Sir James Parks in 1976 remains the standard in surgical practice and forms the basis for understanding the anatomical relationship of fistulous tracts to the anal sphincter complex [8].

Perianal fistulas are generally categorized as simple or complex according to their anatomical course and the extent of anal sphincter involvement [8]. Simple fistulas comprise intersphincteric and low transsphincteric types, whereas complex fistulas include high transsphincteric, suprasphincteric, extrasphincteric, recurrent, and multiple-tract variants. Precise identification of the fistulous tract is essential for selecting the appropriate surgical approach while minimizing the risks of recurrence and sphincter injury [4,9]. According to Goodsall's rule, external openings situated posterior to the transverse anal line typically follow a curved tract that enters the anal canal through the posterior midline, whereas anterior openings usually extend in a radial direction [6]. Although exceptions to this rule are well recognized, it continues to serve as a useful clinical guide for predicting the course of fistulous tracts. In addition, imaging techniques such as magnetic resonance imaging (MRI) and endoanal ultrasonography have significantly improved preoperative evaluation by accurately defining fistulous anatomy, particularly in recurrent and complex fistulas [10,11].

Spontaneous healing of fistula-in-ano is uncommon and surgery remains the definitive treatment modality [9,12]. The ideal surgical procedure should eradicate the fistulous tract, prevent recurrence, and preserve anal sphincter function. Conventional procedures such as fistulotomy and fistulectomy are effective and widely practiced; however, they may be associated with postoperative pain, delayed wound healing, prolonged hospital stay, recurrence, and varying degrees of fecal incontinence due to sphincter damage [13,14]. The risk of incontinence remains a major concern, particularly in procedures involving division of sphincter muscles. Several studies evaluating conventional fistula surgery have highlighted the importance of balancing eradication of disease with preservation of continence [15,16].

Over the past decade, sphincter-preserving techniques for the treatment of perianal fistula, including fibrin glue injection, fistula plugs, video-assisted anal fistula treatment (VAAFT), and ligation of intersphincteric fistula tract (LIFT), have attracted considerable attention as alternatives to conventional procedures [16-18]. The LIFT procedure, first described by Rojanasakul, is based on ligation and division of the fistulous tract within the intersphincteric plane while preserving sphincter integrity [19,20]. The procedure has gained popularity due to its technical simplicity, minimal tissue injury, shorter healing time, and lower risk of postoperative incontinence. Subsequent studies and meta-analyses have reported favorable outcomes with LIFT in terms of postoperative recovery, continence preservation, and recurrence rates [21].

The present study was designed as a prospective, parallel-group randomized controlled trial to compare the clinical outcomes of the LIFT procedure with conventional fistulectomy in patients with perianal fistula. The primary outcomes were postoperative pain, wound infection, and wound healing, while the secondary outcomes included postoperative fecal incontinence and recurrence during follow-up.

## Materials and methods

Ethical approval and trial registration 

Prior to initiating the study, ethical clearance was obtained from the Institutional Ethics Committee of Madras Medical College, Chennai, India (Approval No. 19062022; June 15, 2022). The study protocol was subsequently registered with the Clinical Trials Registry of India under registration number CTRI/2022/06/085935.

Trial design and setting

Utilizing a superiority framework, this research was structured as a prospective, parallel-group, single-center RCT with a 1:1 allocation ratio. The study was conducted in the Department of General Surgery at Chennai's Rajiv Gandhi Government General Hospital, a tertiary care center, between June and December 2022. Conventional fistulectomy was selected as the comparator because it was the routinely performed surgical procedure for eligible patients with perianal fistula at our institution during the study period. This enabled comparison of the LIFT procedure with the prevailing institutional standard of care within the same clinical setting.

Inclusion and exclusion criteria

Eligible participants were adults aged 18 years or older who presented to the Department of General Surgery, Madras Medical College, Chennai, with a confirmed diagnosis of perianal fistula. Several exclusion criteria were applied, which included patients who were under 18, those who withheld consent, or individuals with an immunocompromised status or major medical comorbidities. The study also excluded anyone with a background of tuberculosis, malignancy, or inflammatory bowel disease, as well as those presenting with recurrent or complex perianal fistulas.

Interventions

All participants underwent a comprehensive preoperative assessment that included clinical history, physical examination, and relevant laboratory investigations. Clinical examination served as the primary modality for preoperative evaluation. Magnetic resonance imaging (MRI) was performed selectively in patients with suspected complex fistulas or when the fistula anatomy was uncertain, whereas endoanal ultrasonography was not routinely used. Following the initial evaluation, patients were randomly assigned in a 1:1 ratio to two treatment groups, each comprising 15 participants. Patients allocated to Group A underwent the LIFT procedure, whereas those assigned to Group B received conventional fistulectomy.

Preoperative bowel preparation was carried out using an enema for all participants. A prophylactic dose of 1 g intravenous ceftriaxone was administered before surgery. After the procedure, patients received intravenous ceftriaxone (1 g twice daily) along with intravenous metronidazole (500 mg three times daily) for three days. Analgesics and standard postoperative wound care were provided according to clinical requirements. Patients were subsequently followed up to evaluate postoperative pain, wound healing, continence status, and recurrence.

Outcomes

The outcomes of both procedures were compared based on postoperative pain, wound healing, continence status, and recurrence rates. Postoperative pain was evaluated using the Visual Analogue Scale (VAS), in which patients rated their pain on a scale ranging from zero to ten, with zero indicating no pain and ten indicating the worst possible pain. Wound healing was assessed clinically based on the condition of the surgical wound, presence of granulation tissue, discharge, and complete epithelialization. Continence status was evaluated clinically by assessing the presence or absence of fecal incontinence during follow-up visits. Recurrence was defined as the reappearance of symptoms such as persistent discharge, abscess formation, or redevelopment of a fistulous tract during the follow-up period. Follow-up assessments were performed at one month, three months, and six months to evaluate the parameters mentioned.

Sample size 

The required sample size was estimated using the formula for comparing two independent means, with postoperative pain assessed by the VAS serving as the primary endpoint. Estimates from previously published studies (mean VAS: 4.2 ± 1.3 for LIFT and 6.8 ± 1.5 for conventional surgery) were used for the calculation. Assuming a two-sided significance level of 5% and a statistical power of 80%, the minimum required sample size was estimated to be approximately 13 patients per group. To compensate for potential participant dropout during follow-up, enrolment was increased to 15 patients in each arm, resulting in a total study population of 30.

Randomization

Participants were randomly allocated in a 1:1 ratio to the LIFT or conventional fistulectomy group using a computer-generated random sequence prepared by an independent analyst. Eligible participants were enrolled by the principal investigator. Allocation concealment was ensured using sequentially numbered, sealed opaque envelopes prepared according to the randomization sequence and handled by a research assistant. The envelopes were opened only after participant enrolment to determine treatment allocation.

Statistical analysis 

Data analysis was performed using IBM SPSS Statistics for Windows, version 23.0 (IBM Corp., Armonk, NY, USA). Continuous data were summarized as mean ± standard deviation, while categorical variables were reported as frequencies and percentages. Between-group comparisons were performed using the Mann-Whitney U test for continuous variables. For categorical variables, Fisher's exact test was used because of the small sample size and low event frequencies. Statistical significance was defined as a two-sided p value of <0.05.

## Results

**Figure 1:**
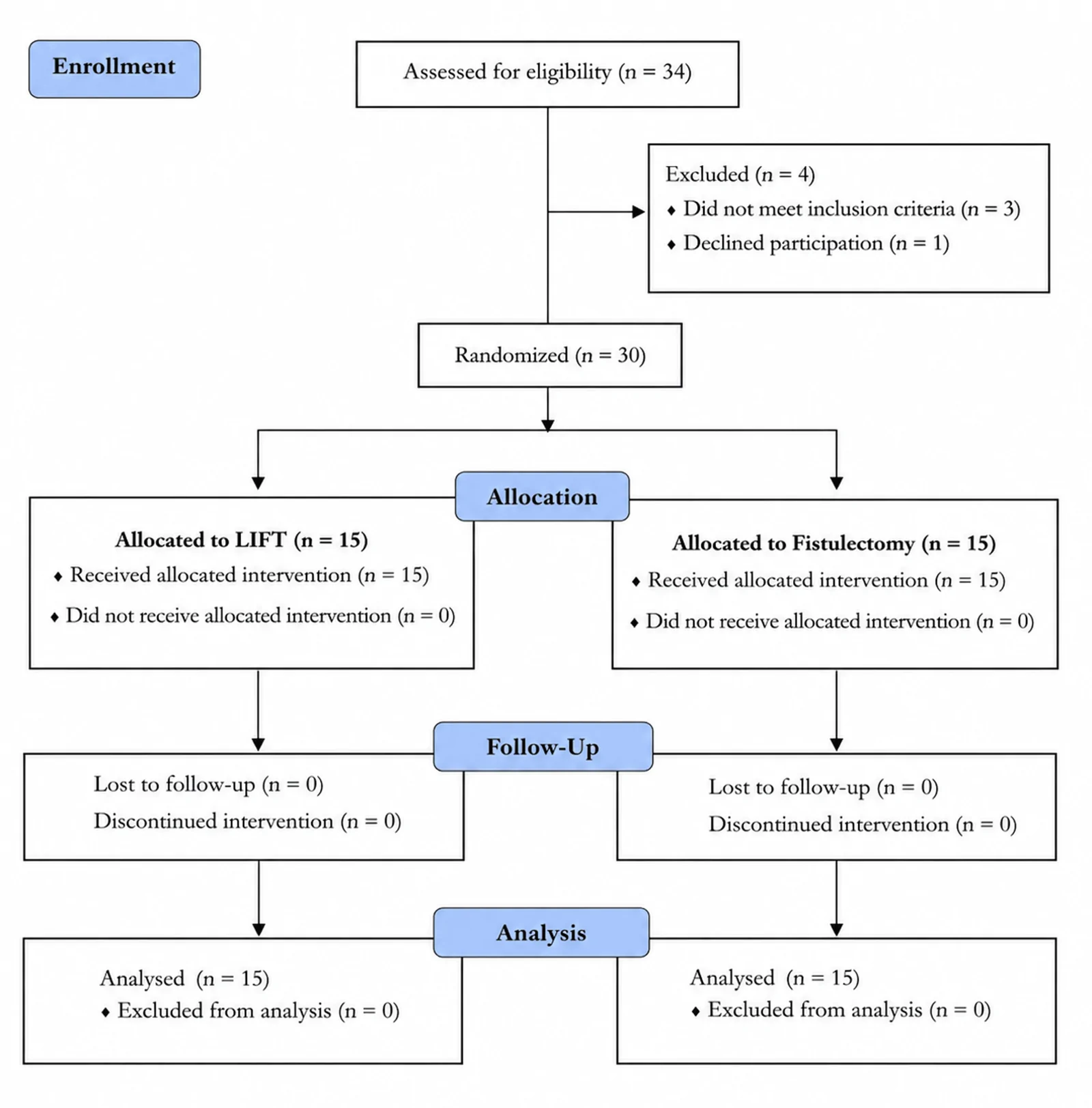
CONSORT 2010 flow diagram of participant enrollment, randomization, follow-up, and analysis LIFT: ligation of intersphincteric fistula tract; CONSORT: Consolidated Standards of Reporting Trials

A total of 34 patients were assessed for eligibility between June and December 2022. Four patients were excluded, including three who did not meet the inclusion criteria and one who declined participation. The remaining 30 eligible participants were randomized in a 1:1 ratio to the LIFT group (n = 15) or the conventional fistulectomy group (n = 15). All randomized participants received the allocated intervention, completed follow-up, and were included in the final analysis (Figure 1).

Most patients were between 40 and 60 years of age, and males constituted the majority of participants in both cohorts. Baseline demographic characteristics are summarized in Table 1.

**Table 1:** Demographic details of the study population LIFT: ligation of intersphincteric fistula tract

Variable	Category	LIFT, n (%)	Fistulectomy, n (%)
Age distribution	<30 years	0 (0.0)	2 (13.3)
	30-40 years	4 (26.7)	3 (20.0)
	40-50 years	4 (26.7)	5 (33.3)
	50-60 years	4 (26.7)	5 (33.3)
	>60 years	3 (20.0)	0 (0.0)
Gender distribution	Male	10 (66.7)	9 (60.0)
	Female	5 (33.3)	6 (40.0)

Patients who underwent the LIFT procedure reported significantly lower postoperative pain than those treated with conventional fistulectomy. Most patients in the LIFT group had VAS scores between 1 and 3, whereas scores ranging from 4 to 7 were more frequently observed among patients who underwent fistulectomy. The mean postoperative VAS score was 2 in the LIFT group compared with 5 in the fistulectomy group, demonstrating a statistically significant difference between the two treatment groups (p = 0.02). A detailed comparison of postoperative pain scores is presented in Table 2.

**Table 2:** Comparison of postoperative pain between study groups LIFT: ligation of intersphincteric fistula tract Statistical significance: p = 0.02 (Mann-Whitney U test)

Visual Analogue Score (VAS)	LIFT, n (%)	Fistulectomy, n (%)
1	4 (26.7)	0 (0.0)
2	5 (33.3)	0 (0.0)
3	4 (26.7)	2 (13.3)
4	1 (6.7)	3 (20.0)
5	1 (6.7)	5 (33.3)
6	0 (0.0)	3 (20.0)
7	0 (0.0)	2 (13.3)

Postoperative wound infection was less frequent in the LIFT group than in the conventional fistulectomy group (6.7% vs. 46.7%). This difference was statistically significant (Fisher's exact test, p = 0.035), with patients undergoing LIFT demonstrating a lower relative risk of postoperative wound infection (RR = 0.416, 95% CI: 0.225-0.766). The comparison of postoperative wound infection between the study groups is presented in Table 3.

**Table 3:** Comparison of postoperative wound infection between study groups LIFT: ligation of intersphincteric fistula tract Statistical significance: p = 0.035 (Fisher's exact test)

Wound infection	LIFT, n (%)	Fistulectomy, n (%)
No	14 (93.3)	8 (53.3)
Yes	1 (6.7)	7 (46.7)

Patients who underwent the LIFT procedure demonstrated more favorable postoperative wound healing than those treated with conventional fistulectomy. Good wound healing was achieved in 93.3% of patients in the LIFT group compared with 53.3% in the conventional fistulectomy group. This difference was statistically significant (Fisher's exact test, p = 0.035), with the LIFT group demonstrating a significantly lower risk of poor wound healing (RR = 0.416, 95% CI: 0.225-0.766). The comparison of postoperative wound healing between the study groups is presented in Table 4.

**Table 4:** Comparison of postoperative wound healing between study groups LIFT: ligation of intersphincteric fistula tract Statistical significance: p = 0.035 (Fisher's exact test)

Wound healing	LIFT, n (%)	Fistulectomy, n (%)
Good	14 (93.3)	8 (53.3)
Poor	1 (6.7)	7 (46.7)

Throughout the follow-up period, patients treated with the LIFT procedure experienced fewer recurrences than those who underwent conventional fistulectomy. Over a period of six months, recurrence occurred in 6.7% of patients in the LIFT group compared with 26.7% in the conventional fistulectomy group. Although the LIFT group demonstrated a lower risk of recurrence (RR = 0.550, 95% CI: 0.295-1.03), the difference was not statistically significant (Fisher's exact test, p = 0.330). Detailed recurrence outcomes are presented in Table 5.

**Table 5:** Comparison of recurrence between study groups LIFT: ligation of intersphincteric fistula tract Statistical significance: p = 0.330 (Fischer's exact test)

Follow-up period	LIFT recurrence, n (%)	Fistulectomy recurrence, n (%)
1 month	1 (6.7)	1 (6.7)
3 months	0 (0.0	2 (13.3)
6 months	0 (0.0)	1 (6.7)
Total recurrence	1 (6.7)	4 (26.7)

No postoperative fecal incontinence was observed in the LIFT group, whereas transient postoperative fecal incontinence occurred in one patient (6.7%) in the conventional fistulectomy group. The difference between the groups was not statistically significant (Fisher's exact test, p = 1.000). The comparison of postoperative fecal incontinence is presented in Table 6.

**Table 6:** Comparision of postoperative fecal incontinence between study groups LIFT: ligation of intersphincteric fistula tract Statistical significance: p = 1.000 (Fisher's exact test)

Fecal incontinence	LIFT, n (%)	Fistulectomy, n (%)
No	15 (100.0)	14 (93.3)
Yes	0 (0.0)	1 (6.7)

Effect size analysis demonstrated lower risks of adverse postoperative outcomes among patients undergoing the LIFT procedure, as shown in Table 7. The risk of postoperative wound infection was lower in the LIFT group than in the conventional fistulectomy group (RR = 0.416, 95% CI: 0.225-0.766). Similarly, the risk of poor wound healing was lower following LIFT (RR = 0.416, 95% CI: 0.225-0.766). The risk of recurrence during follow-up was also lower in the LIFT group (RR = 0.550, 95% CI: 0.295-1.03); however, this difference was not statistically significant. Overall, the effect estimates favored the LIFT procedure, although the findings should be interpreted with caution given the small sample size and the limited precision of some estimates.

**Table 7:** Effect size estimates for major postoperative outcomes LIFT: ligation of intersphincteric fistula tract An RR <1 indicates a lower risk of the specified outcome in the LIFT group relative to the fistulectomy group.

Outcome	LIFT n/N (%)	Fistulectomy n/N (%)	Risk ratio (RR)	95% confidence interval
Wound infection	1/15 (6.7)	7/15 (46.7)	0.416	0.225-0.766
Poor wound healing	1/15 (6.7)	7/15 (46.7)	0.416	0.225-0.766
Recurrence	1/15 (6.7)	4/15 (26.7)	0.550	0.295-1.03

## Discussion

The majority of perianal fistulas result from cryptoglandular infection involving the anal glands in the intersphincteric plane. Their classification is based on the anatomical relationship of the fistulous tract to the anal sphincter complex, as outlined by Parks et al. [8]. This study aimed to compare the clinical outcomes of the LIFT procedure with those of conventional fistulectomy in patients with perianal fistula by evaluating postoperative pain, wound healing, postoperative complications, and recurrence. An optimal procedure for fistula-in-ano should ensure complete elimination of the fistulous tract without compromising anal sphincter function, while also minimizing the risk of recurrence. While traditional methods like fistulotomy and fistulectomy are effective, they still present concerns regarding postoperative morbidity and the potential for sphincter injury [4,9]. In recent years, sphincter-preserving procedures have gained acceptance due to their potential to reduce postoperative complications while maintaining continence [15]. The LIFT procedure, first described by Rojanasakul et al., is based on ligation and division of the fistulous tract within the intersphincteric plane, thereby avoiding significant injury to the external anal sphincter [19]. Previous studies have reported favorable outcomes with LIFT in terms of reduced postoperative pain, faster wound healing, preservation of continence, and acceptable recurrence rates [21-23]. Based on these principles, the present study compared LIFT and fistulectomy using postoperative pain scores, wound infection, wound healing, and recurrence during follow-up as the primary outcome measures.

The study cohort comprised 30 patients diagnosed with perianal fistula, the majority of whom were aged 30-60 years. A slight predominance of male patients was noted in both study groups. These findings are comparable with the epidemiological observations reported by Sainio P, who noted a higher incidence of perianal fistula among middle-aged individuals with male predominance [24]. Similar demographic patterns were also described by Seow-Choen et al. and Hamalainen et al., who reported that fistula-in-ano commonly affects economically active adults and occurs more frequently in males [1,3]. The observed male predominance in the present study may be attributed to the higher incidence of cryptoglandular infections and anorectal sepsis among men. 

In the present study, patients who underwent the LIFT procedure experienced significantly lower postoperative pain scores than those treated with conventional fistulectomy. The mean postoperative VAS in the LIFT group was lower than that of the fistulectomy group, and the average wound healing time was also shorter following LIFT. This may be attributed to the functional preservation nature of the procedure, minimal tissue handling, and the relatively smaller operative wound associated with LIFT. In comparison, fistulectomy requires complete excision of the tract with a larger raw area, thereby increasing postoperative discomfort and delaying wound healing. Similar observations have been reported by Mushaya et al., who noted improved postoperative recovery with LIFT when compared to other conventional procedures [21]. Blumetti et al. also emphasized the growing preference for limited sphincter disruption procedures due to their lower postoperative morbidity and improved functional outcomes [15].

Postoperative wound infection was less frequent in the LIFT group than in the conventional fistulectomy group. This finding is consistent with previous reports suggesting that the limited tissue dissection associated with LIFT reduces postoperative wound contamination and facilitates wound healing. The lower risk of wound infection observed in the present study further supports the role of sphincter-preserving procedures in reducing postoperative morbidity.

A lower incidence of postoperative fecal incontinence and recurrence was observed among patients who underwent the LIFT procedure compared with those treated by conventional fistulectomy. No patient in the LIFT group developed postoperative fecal incontinence, whereas transient postoperative fecal incontinence was observed in one patient following conventional fistulectomy. Likewise, recurrence occurred less frequently following the LIFT procedure. However, neither postoperative fecal incontinence nor recurrence demonstrated a statistically significant difference between the study groups, and these findings should therefore be interpreted cautiously. Preservation of the anal sphincter complex remains one of the principal goals in the surgical management of fistula-in-ano, as even minor sphincter injury can adversely affect long-term functional outcomes and quality of life. Because the LIFT procedure involves ligation of the fistulous tract within the intersphincteric plane without division of the external anal sphincter, the risk of continence disturbance is theoretically reduced. Shanwani et al. reported favorable continence outcomes with LIFT and demonstrated its feasibility as a sphincter-preserving technique [23]. Similar observations were reported by Sileri et al., who demonstrated satisfactory healing rates with minimal impairment of continence following LIFT [25].

Recurrence remains one of the most challenging aspects in the management of perianal fistula. In the present study, recurrence occurred less frequently following the LIFT procedure than after conventional fistulectomy; however, the difference was not statistically significant. Adequate identification and ligation of the intersphincteric tract, together with eradication of the infected cryptoglandular focus, may contribute to the lower recurrence rates observed following LIFT. Hong et al., in their systematic review and meta-analysis, reported acceptable healing and recurrence rates with preservation of sphincter function following LIFT [26]. Nevertheless, recurrence rates reported in the literature remain variable and are influenced by factors such as fistula complexity, patient selection, surgeon experience, and duration of follow-up. Accordingly, the recurrence findings of the present study should be interpreted cautiously in view of the relatively small sample size and limited follow-up period.

The findings of the present study are generally consistent with current international recommendations regarding sphincter-preserving procedures for cryptoglandular anal fistula. Recent systematic reviews and meta-analyses, together with the American Society of Colon and Rectal Surgeons (ASCRS) Clinical Practice Guidelines, recognize the LIFT procedure as an effective sphincter-preserving option for appropriately selected patients because of its favorable healing rates and preservation of continence [27]. Nevertheless, treatment should be individualized according to fistula anatomy, patient characteristics, and surgeon expertise. The findings of the present study are broadly consistent with these contemporary recommendations.

The findings of the present study suggest that the LIFT procedure may be associated with favorable postoperative outcomes compared with conventional fistulectomy in the management of perianal fistula. Patients undergoing LIFT demonstrated significantly lower postoperative pain scores, lower rates of postoperative wound infection, and improved wound healing. Although postoperative recurrence and fecal incontinence occurred less frequently following the LIFT procedure, these differences were not statistically significant. Overall, these findings should be interpreted cautiously because of the relatively small sample size, single-center study design, and limited follow-up duration.

The present study should be interpreted in light of certain limitations. The relatively small sample size of 30 patients may limit the generalizability of the findings. Additionally, the follow-up period was limited, precluding a comprehensive assessment of long-term recurrence and continence outcomes. The anatomical classification of fistulas according to the Parks classification was not consistently documented in the available records [8]. Consequently, the distribution of intersphincteric and transsphincteric fistulas between the study groups could not be reported, limiting the assessment of baseline anatomical comparability. Furthermore, as this was a single-center study conducted at a tertiary care institution, the findings may have been influenced by institutional practices and surgeon-related factors. Future multicenter studies involving larger patient populations and longer follow-up periods are warranted to further evaluate the efficacy, safety, and long-term outcomes of the LIFT procedure compared with conventional fistulectomy.

## Conclusions

The present study suggests that the LIFT procedure is an effective sphincter-preserving option in the management of perianal fistula. Compared with conventional fistulectomy, patients undergoing LIFT experienced significantly lower postoperative pain scores, lower rates of postoperative wound infection, and improved wound healing. Although postoperative fecal incontinence and recurrence occurred less frequently following LIFT, these differences did not reach statistical significance.

Overall, the findings indicate that LIFT is a safe and promising alternative to conventional fistulectomy in appropriately selected patients; however, they should be interpreted cautiously in view of the relatively small sample size, limited follow-up period, and single-center study design. Larger multicenter studies with longer follow-up are warranted to further validate these findings.
